# From the Editors

**DOI:** 10.35946/arcr.v40.2.14

**Published:** 2020-11-05

**Authors:** Mary E. McCaul, Ivana Grakalic

**Affiliations:** 1 Department of Psychiatry and Behavioral Sciences, Johns Hopkins University School of Medicine, Baltimore, Maryland; 2 Division of Neuroscience and Behavior, National Institute on Alcohol Abuse and Alcoholism, National Institutes of Health, Bethesda, Maryland

Recent epidemiological research has identified alarming trends in drinking patterns of girls and women in the United States. In recent years, the amount and frequency of alcohol use are increasing in White and Hispanic girls and young women in contrast to decreasing patterns of heavy alcohol use in boys and young men.^1^,^2^ Similarly, current and binge alcohol use is rising among older women,^3^,^4^ resulting in increased morbidity and mortality in this growing segment of the U.S. population. For example, emergency room visits associated with both acute and chronic drinking^5^ and alcohol-related inpatient diagnoses in U.S. middle-aged adults^6^ have accelerated more rapidly in women than men. Overall, these changes have narrowed the long-established gender gap in alcohol consumption and associated problems, with women’s drinking patterns across the life cycle approaching those of men.

These epidemiological trends have increased the urgency of sex-specific, gender-focused research on alcohol.^7^ Historically, because they were underrepresented among heavy/problem drinkers, women often were omitted from a wide range of alcohol studies, including basic science on alcohol effects in women, alcohol-related medical morbidities, social/behavioral consequences of drinking, and treatment intervention studies. With this topic series on women and alcohol, *Alcohol Research: Current Reviews (ARCR)* seeks to close these knowledge gaps and identify important areas for future research directions.

“Gender Differences in the Epidemiology of Alcohol Use and Related Harms in the United States” provides an update on the diminishing sex differences in alcohol consumption, related health problems, hospitalizations, emergency department visits, and death across the life span.^8^ Of particular concern, White highlights the reversal in historical alcohol consumption patterns of underage drinkers, such that adolescent girls now report higher rates of monthly alcohol use and binge drinking compared with adolescent boys.^8^ Findings have important implications for prevention of fetal alcohol spectrum disorders.

As illustrated in articles throughout this *ARCR* topic series, many alcohol-related sex differences—including development and maintenance of alcohol misuse, alcohol-driven cognitive and medical problems, and even psychiatric comorbidities—derive from key differences in the neurobiology of men and women. In “Sex Differences in the Neurobiology of Alcohol Use Disorder,” Flores-Bonilla and Richardson explore preclinical and human research on neural differences using a three-stage framework of addiction.^9^ Specifically, they examine how neurobiological differences contribute to initial development of binge/intoxicated drinking, the transition into withdrawal, negative affect and dysfunctional behaviors associated with continued heavy drinking, and finally development of preoccupation with or craving for alcohol and compulsive drinking, and relapse.^9^

In “The Endocrine System and Alcohol Drinking in Females,” Finn extends this neurobiological review by examining the multidirectional interactions of alcohol, stress, and key gonadal sex steroid hormones and stress steroid hormones.^10^ Findings suggest promising directions for development of novel pharmacological treatments for alcohol use disorder (AUD).

In “Alcohol’s Unique Effects on Cognition in Women: A 2020 (Re)view to Envision Future Research and Treatment,” Fama, Le Berre, and Sullivan provide a wide-ranging update on the interrelationships between alcohol and cognition, including effects of acute and chronic alcohol consumption across the drinking continuum.^11^ Although current research indicates many overall similarities in structural and functional effects of alcohol in women and men, the authors bring focus to factors that may influence sex-specific differences, such as age, drinking patterns, abstinence duration, and medical history and psychiatric comorbidities.^11^ One area of particular relevance for women is the effects of alcohol on social and emotional cognition; this relatively young area of cognitive research has important implications for both development and consequences of AUD. Overall, it is clear that women who are chronic heavy drinkers experience cognitive deficits relative to age-matched women who are social drinkers or do not drink. These findings should be used to inform development and adaptations of alcohol treatment interventions and recovery programs for women.

It is well established that women experience higher prevalence of mood and anxiety disorders^12^ and more frequent interpersonal trauma associated with higher prevalence of post-traumatic stress disorder^13^ compared with men, and that these negative factors have a role in the development and maintenance of heavy drinking and associated problems in women. In “The Role of Stress, Trauma, and Negative Affect in the Development of Alcohol Misuse and Alcohol Use Disorder in Women,” Barros Guinle and Sinha examine the sex-specific neurobiological underpinnings of the biological, psychosocial, and psychiatric factors that may be contributing to the accelerating drinking patterns recently observed in girls and women.^14^ Of particular concern is the growing evidence of a sex-related, chronic negative feedback cycle in which childhood maltreatment and trauma lead to the development of a maladaptive, blunted stress response in girls and women.^14^ In turn, this blunted neurobiological response escalates alcohol consumption, further blunting neuroendocrine responses, and contributing to the progression from alcohol misuse to AUD.

Given differences between women and men in risk factors, developmental course, and health and psychosocial consequences of alcohol misuse and AUD, tailored approaches to alcohol identification, prevention, and intervention for girls and women may be necessary to maximize treatment outcomes. Indeed, specialized screening instruments that are more sensitive and specific to women are available to improve case identification.^15^ Although evidence suggests that women and men have comparable outcomes in mixed-gender, nonspecialized alcohol treatments,^16^ women cared for in specialized, women-specific programs may experience greater improvements in key areas such as pregnancy outcomes, psychiatric health, HIV risk reduction, and psychosocial well-being.^17^ These areas are reviewed in several key articles in this topic series.

In “Maternal Substance Use: Consequences, Identification, and Interventions,” Chang reviews prevalence and addresses the importance of early identification and intervention for substance use among pregnant women, with emphasis on alcohol, tobacco, cannabis, and opioid exposure.^18^ She reviews strengths and shortcomings of available screening tools specific to pregnant women, legal and social barriers to implementation of universal screening, and available prevention intervention strategies, particularly for fetal alcohol spectrum disorders.^18^

In “Alcohol Screening, Brief Intervention, and Referral to Treatment (SBIRT) for Girls and Women,” Hammock, Velasquez, Alwan, and von Sternberg provide a comprehensive review of the effectiveness of this evidence-based, public health approach to identifying and intervening in heavy/harmful alcohol use across the life span, specifically examining SBIRT for girls, women of childbearing age, and older women.^19^ This clinically relevant, evidence-based article offers information on age-appropriate screening tools and intervention approaches.^19^ It also summarizes facilitators and barriers to SBIRT implementation in social service and health care settings,^19^ including recently identified unanticipated consequences of state-level policies related to alcohol use during pregnancy.^20^

“Treatment Interventions for Women With Alcohol Use Disorder” examines women’s barriers to treatment seeking and referral, program services to address these barriers, and efficacy of women-specific services relative to traditional mixed-gender care.^21^ Importantly, McCrady, Epstein, and Fokas address mechanisms of change, which often are overlooked but highly relevant to successful development of strategies to tailor treatment to women more effectively.^21^ Finally, the article considers the effects of women-specific substance abuse services on a breadth of outcomes, ranging from the primary targets of alcohol and drug use to secondary outcomes such as psychosocial well-being, psychiatric health, pregnancy outcomes, and HIV risk reduction.^21^

Although much of the research discussed in this topic series addresses sex-specific findings, it is critical to bear in mind that this literature often obscures important differences among women as a group. In “Alcohol-Related Disparities Among Women: Evidence and Potential Explanations,” Mulia and Bensley address key foci of diversity research, including race, ethnicity, socioeconomic and social status, and sexual orientation.^22^ Although the research to date is quite limited, these factors have been shown to influence not only effects of acute and chronic alcohol consumption, but also alcohol-related health disparities and access to care. The article highlights the “alcohol harm paradox”^23^—that certain racial/ethnic minority groups, particularly African Americans, and lower socioeconomic groups experience greater harm despite comparable or lower alcohol consumption. The authors consider possible explanations and interventions for these disparities.^22^

Finally, we have known for decades that women are more vulnerable to many of the negative health consequences of alcohol consumption, in part, due to their higher blood alcohol levels achieved at comparable alcohol doses compared with men. Now, research is providing system-specific findings of the interplay of alcohol and health in women. Indeed, this topic series addresses sex-specific health effects of alcohol in four key areas. In “Alcohol and Liver Function in Women,” Maddur and Shah address the increasing rates of liver disease in women, the key role that estrogen plays in the greater vulnerability and more rapid progression to alcohol-related liver disease in women compared with men, and sex differences in liver transplant availability and outcomes.^24^

In “Alcohol’s Effects on Breast Cancer in Women,” Freudenheim highlights the compelling evidence that any alcohol use increases breast cancer risk and that risk increases as total consumption increases, emphasizing the importance of targeting this modifiable risk factor for public education and intervention.^25^ Current findings suggest that these effects are independent of alcohol beverage type or age at alcohol exposure. The author reviews possible mechanisms for this increased risk including direct carcinogenic effects of alcohol and acetaldehyde, changes in hormones associated with drinking, and alterations in DNA methylation.^25^

Cardiovascular (CV) diseases (e.g., hypertension, coronary heart disease, stroke) are the leading cause of death in women.^26^ In “Effects of Alcohol on the Cardiovascular System in Women,” Piano, Thur, Hwang, and Phillips address the sex-specific findings about the contribution of alcohol consumption to CV morbidity and mortality.^27^ Unlike the generally linear relationship between drinking and CV disease in men, there appears to be a J-shaped function for women, with no or lower CV risk at one or two drinks per day and increased risk at and above three or four drinks per day.^27^ The authors examine the contributions of estrogen to these relationships.^27^

Women are more likely to experience insomnia and other common forms of sleep dysregulation compared with men and, in turn, sleep disruption has more severe health consequences for women compared with men.^28^ Despite the fact that sleep disturbance is one of the most frequent complaints among persons with AUD,^29^ sex differences in sleep have been understudied and underreported in alcohol research. In “Sleep and Alcohol Use in Women,” Inkelis, Hasler, and Baker consider important bidirectional effects of alcohol and sleep disruption, examining both how poor sleep quality may contribute to alcohol consumption and how acute and chronic alcohol consumption can lead to sleep dysregulation.^30^ The authors review biological, psychological, and social factors that contribute to these bidirectional relationships as well as their treatment implications.^30^

All of the articles in this topic series highlight critical, ongoing, sex-specific knowledge gaps in our understanding of the epidemiology of alcohol use, the interplay of physiology and alcohol, and best approaches to prevention and treatment. This research supports the importance of the National Institutes of Health mandate not only to include female subjects in research, but also to include them in sufficient numbers to permit sex-specific analyses of findings. As evidenced by these articles, the National Institute on Alcohol Abuse and Alcoholism has successfully targeted many of these areas for support in recent years, yet much remains to be learned as we confront the rapidly changing characteristics of women’s alcohol misuse and harms.
